# Preference for care models among older people living with HIV: cross-sectional study

**DOI:** 10.1186/s12889-023-16941-9

**Published:** 2023-10-18

**Authors:** Mei Li, Jianlan Ren, Yue Luo, Roger Watson, Yu Zheng, Li Ding, Fulan Wang, Yanhua Chen

**Affiliations:** 1 Department of Gynecology, The First Branch of The First Affiliated Hospital of Chongqing Medical University, Chongqing, China; 2 Department of Anesthesiology, The Affiliated Hospital of Southwest Medical University, Luzhou, China; 3 Department of Cardiology, West China Hospital, Sichuan University, Chengdu, China; 4 Health and Social Care Faculty, University of Hull, Hull, UK; 5 Department of Rheumatism and Immunology, The Affiliated Hospital of Southwest Medical University, Luzhou, China; 6 Department of Gynecology, The First Affiliated Hospital of Chongqing Medical University, Chongqing, China; 7 Department of Nursing, The Affiliated Hospital of Southwest Medical University, Luzhou, China

**Keywords:** Older, Care models, Preference, HIV stigma, Social support

## Abstract

**Background:**

The number of people living with HIV (PWH) aged 50 and above is increasing. The question of care among older PWH (aged ≥ 50 years) is an increasing concern. Understanding the care preference of older people can better provide care services for them. The purpose of this study was to investigate the care preference (home-based care, self-care, institutional care, community-based care, and mutual-aid care) among older PWH and identify the factors affecting their care preference.

**Methods:**

A cross-sectional survey was conducted among older PWH (aged ≥ 50 years) about care preference from May to November 2021. We enrolled 319 participants using convenience sampling. We designed a questionnaire to assess the care preference of older PWH. The Chi-square test and Fisher’s exact test were used to conduct univariate analysis of care preference. Multinomial logistic regression was used to identify factors influencing care preference.

**Results:**

Most older PWH (72.7%) preferred home-based care, and few (15.7%) preferred self-care. Fewer older PWH preferred community-based care (5.3%), institutional care (5.0%) and mutual-aid care (1.3%). Multivariate analysis showed that older PWH with a house, spouse and more children were more inclined to choose home-based care (*p* < .05). Older PWH living alone, having higher monthly income and higher HIV stigma preferred to choose self-care (*p* < .05).

**Conclusion:**

Home-based care was the most preferred model of older PWH, and self-care ranked second. The number of those who preferred institutional care, community-based care and mutual-aid care were few. Nation and government should take measures to allocate care resources for older adults reasonably to better meet the care needs of older PWH. It is important to strengthen social security, reduce internalized HIV stigma, improve social support, and explore diversified care models for improving the quality of life of older PWH.

**Supplementary Information:**

The online version contains supplementary material available at 10.1186/s12889-023-16941-9.

## Introduction

Health and aging have become important global issues because of the aging population. In the field of AIDS, older people living with HIV (PWH) are usually defined as ≥ 50 years old in order to distinguish them from sexually active groups (15–49 years) [1]. In addition, older PWH on Highly Active Antiroviral Therapy (HAART) show worse immune recovery than younger people with HIV, often with immune failure and accelerated immune aging. Other age-related diseases (geriatric syndromes, functional or neurocognitive/mental health problems, and polymedication) also have higher prevalence and poorer survival rates than uninfected people [2, 3]. Therefore, the international community usually defines age ≥ 50 as the medical age of people with HIV, and PWH over 50 years old are the elderly [1, 4, 5]. HAART has reduced mortality among PWH, resulting in an aging HIV population [3]. In addition, this can be attributed to the increase in the number of PWH over the age of 50 [5]. UNAIDS reported in 2021 that 8.1 million people aged 50 and over worldwide had HIV. One modelling study [ 6] conducted in the Netherlands found that the proportion of PWH aged 50 years or older will reach three-quarters by 2030. According to China CDC Weekly [7], the proportion of newly infected males aged 60 and above increased to 18.21% in 2020. Older PWH represents the fastest growing group among the HIV-infected patients in the UK, with one-third of PWH over the age of 50 [8]. In view of the growing burden of older PWH, old age care in this segment of the population is on the agenda.

Research has characterized how the social support of older PWH may be characterized as inadequate, given the lack of family involvement, poor perceived availability and high degrees of social isolation [9]. Affected by old age and HIV, older PWH are considered vulnerable and they may require support from family and society [10]. Family is an important informal support resource that provides emotional and instrumental support for older PWH [11, 12]. However, there was widespread social isolation and low family support among older PWH due to a number of factors such as internalized HIV stigma, public discrimination and poverty. Simultaneously, HIV stigma and age discrimination made older PWH separate from informal support networks [13, 14]. One study [15] found that more than 70% of older PWH lived alone, and only 15% had a lifetime partner. Another study pointed out that social isolation was common among older PWH [14]. Other studies [16, 17] found that only one-third of older PWH had a partner and 71% lived alone.

According to Cantor’s Hierarchical Compensatory Theory of Social Support, the network fragility of older PWH can drive their formal service use [9]. Thus older PWH may seek home and community-based care services [18]. In sub-Saharan African countries, PWH can receive medical care at home, and community-based care volunteers and home-based caregivers provide daily care and primary care [19]. In Australia, PWH could access to specialist government-funded services through the Community HIV Nursing Program [20]. The types of services or support included health monitoring, medication management, care coordination, mental health support, financial/housing support and so on. Many PWH have received community HIV nursing services for 2 to 13 years. In British Columbia (BC), Canada, the government has established a publicly funded home and community-based care (HCC) system, which has provided formal support services such as home support, community-based care, community rehabilitation and residential care for 15% of older PWH [ 21]. However, due to public funding cuts in HCC home support services, access to home support for seniors in BC has decreased by 30% between 2001 and 2016.

The stigma of HIV has left older PWH with limited traditional support networks, making them more reliant on formal care providers and hence the need for long-term care services for this group [11, 22]. Nursing homes are a key component of the continuum of long-term care available to people with HIV disease, providing a cost-effective alternative for PWH who can no longer receive appropriate care from home and community-based services [23]. However, older PWH were not well-accepted by nursing homes. A survey of 53 nursing homes in France [15] found that nursing home physicians and staff were not yet ready to face the admission of PWH, and only four physicians had admitted PWH to their nursing home. From 1989 to 2000, several surveys on nursing home staff were conducted in the US. Studies [24, 25] found that managers were less willing to accept PWH, and nursing homes tended to avoid the issue of PWH admission. With the continuous improvement of policies and laws in the United States, the acceptance of PWH in nursing homes was improved A study [26] found that between 2001 and 2010 in the US, the prevalence of HIV in nursing homes increased from 0.7 to 1.2% with a 71% increase, and 92493 (1%) of residents had HIV. In China, old age care service institutions do not admit patients with infectious diseases (including PWH). Before entering the nursing home, older people were required to undergo physical examination. Once infectious diseases are found, most nursing homes will refuse older people.

Old-age care is an important livelihood issue. At present, there are few studies about care preference among older PWH are few. Older PWH are a special group of older people whose old-age care is not only about social security, but also about public health. Therefore, the aim of this study was to investigate the care preference of older PWH and identify its influencing factors to provide a reference point for the relevant departments to improve the pension service security system. This study, from the perspective of the care needs and preferences of older PWH, can raise the attention of the public, organizations and society to the problem of older PWH. The results of the study can be used as a reference for similar regions and provide data support and suggestion references for the government to solve the problem of old-age care for this group, to improve their quality of life.

## Methods

### Study design and setting

This was a cross-sectional study. PWH usually received ART follow-up management in the designated medical and health institutions. Therefore, all participants in this study received HIV antiretroviral therapy. Most of the health care facilities for HIV follow-up management will specify the time when medical check-ups and free antiretroviral treatment medication can be received, and PWH should receive their follow-up management at the specified time and place. The CDC is the superior institution that manage these HIV follow up medical facilities and we obtained consent from the CDC prior to entering these facilities.

### Measures

(1) Socio-demographic characteristics: including individual situation (gender, age, education, household registration); family situation (marriage, self-house, number of children, etc.); economic situation (family economic stability, personal income, endowment insurance, etc.); and disease situation (length of diagnosis, chronic diseases, self-rated health, and HIV disclosure).

(2) Care willingness and preference: This was measured by two questions: “Which of the following old-age care models can you accept?” (multiple choices); and “Which old-age care models do you prefer?” (single choice). Both questions have five options (home-based care, community-based care, institutional care, mutual-aid care and self-care). Home-based care refers to the traditional Chinese care model in which old-age care was provided by children or family members. Community-based care means that communities provide older people with door-to-door services, community center services and so on. Institutional care refers to a socialized care model in which old age care institutions provide care services. Mutual-aid care refers to the mutual assistance between older people and other volunteers. Self-care refers to older people mainly relying on themselves economically, in life and in spirit.

(3) Social support: The social support scale developed by Chinese scholar Xiao [27] was used to measure the social support of older PWH and to explore the impact of social support on care preference. It has 10 items, including three dimensions, namely subjective support (score 1–22), objective support (score 8–32) and use of social support (score 3–12). The scale is widely used and has good reliability. The Cronbach’s α coefficient of this original scale is 0.815. The total score of social support ranges from 12 to 66. A higher score indicates a higher level of social support. Low-level social support scores ranged from 12 to 22, medium-level social support scores ranged from 23 to 44, and high-level social support scores ranged from 45 to 66.

(4) HIV stigma: The Simplified Berger HIV Stigma Scale [28] was used to measure the internalized stigma of older PWH and to explore the impact of disease stigma on care preference. The scale was derived from the Berger HIV Stigma Scale. Scholars from Peking University in China simplified the Berger scale and tested the reliability and validity of the SBHSS in 587 HIV-infected patients. The scale has basic reliability and validity and the Cronbach’s α coefficient of the original scale was 0.78. The scale has 15 items, including four dimensions of “rejection of infected persons, negative self-impression, fear of identity exposure and harm for identity exposure”. Each item has two options: “Yes” and “No”. One point was given for “Yes” and 0 points for “No”. The total score of HIV stigma was 15 points, and the higher the score was, the higher the stigma was. According to scores of 0, 1 to 5, 6 to 10 and 11 to 15, HIV stigma was classified into four categories: none; mild; moderate; and severe.

### Participants

The sample size is calculated as a rule of thumb. A good general rule of thumb for factor analysis is 300 cases [29]. Samples were obtained by convenience sampling. In this study, we collected 319 valid samples. The inclusion criteria of this study were as follows: (1) HIV-positive; (2) aged ≥ 50 years; (3) clear consciousness (without known significant cognitive impairment, such as dementia); (4) the ability to communicate well; and (5) informed consent and voluntary participation in the study.

### Data collection

We designed a questionnaire for older PWH based on literature research and expert consultation. We invited five experts engaged in geriatric research or AIDS research to conduct questionnaire consultation. A preliminary investigation was conducted before the formal investigation. According to the problems existing in the preliminary survey, the questionnaire was improved and revised to form the final formal questionnaire. A presurvey of 29 older PWH was conducted to assess the level of understanding of the content of the questionnaire and to test the feasibility of the study methodology [30]. We found that older PWH have some preferences for self-care and a high level of HIV stigma, and there is a need to use the local dialect for the articulation of some issues. Therefore, based on the presurvey, we added self-care to the preferences for old-age care modes, added an AIDS stigma scale, and elaborated and supplemented the definitions of care modes in more detail, so that respondents could understand the content of the survey. In the survey, we used dialects to explain our questions.

Considering the specificity of AIDS, convenience sampling was adopted for this study. Interested or willing participants were invited to participate in this study. From May to November 2021, the study was conducted among older PWH aged 50 and above in the designated medical and health institutions in Luzhou, China. During data collection, we recruited participants on site with the help of the heads of these facilities. The content, purpose, and significance of the study were explained to the study participants before the survey, and their informed consent was obtained. A Chinese paper-based questionnaire was used to collect information from older people living with HIV by trained investigators (they were mainly team members of this study). Those who had a high level of education and could understand the content of the questionnaire were asked to self-filled questionnaire. If the participants do not understand the questions, our investigators provided them with explanations. Those with low education and those unable to fill in the questionnaire were filled out by investigator interview. After the survey was completed, the questionnaires were collected on the spot and checked in a timely manner, and any questionnaires that did not meet the requirements were immediately verified with the respondents and supplemented. A total of 340 questionnaires were distributed. After excluding invalid questionnaires, 319 valid questionnaires were obtained, and the effective recovery rate was 93.8%.

### Statistical analysis

Data were analysed using SPSS 21.0. The continuous variables were subject to normal distribution using the mean and standard deviation (SD). Categorical variables were statistically described by frequency (percentage). According to the differences in the main sources of social support for the care of older people, old age care models were divided into three main categories (home-based care, self-care, and socialized care). According to Chinese scholars [31], socialized old-age care is defined as a form of old-age care that is differentiated from traditional family care and is guaranteed by a social system and social channels. Therefore, socialized care includes institutional care, community-based care, and mutual-aid care. The dependent variable was the three categories of old-age care preferences (home-based care, self-care and socialized care). First, univariate analysis (Chi-square test and Fisher’s exact probability method) was used to assess the influencing factors of care model preference. Then the variables with statistical significance in univariate analysis were included in a multivariate analysis (multinomial logistic regression). Education level, number of children, monthly personal income, family economic stability, length of diagnosis, self-rated health, social support level, and degree of HIV stigma were used as continuous variables in the statistical analysis. Other independent measures were used as categorical variables. Multinomial logistic regression includes two models. In Model 1, self-care was compared with home-based care. In model 2, socialized care was compared with home-based care; P < .05 was considered statistically significant.

## Results

### Characteristics of the respondents

Table 1 presents the demographic information of the participants. There were 319 participants with a mean age of 64.59 (SD ± 8.69 years). Participants in this study were between 50 and 86 years old. Participants were predominantly male (73.0%). Primary education or less accounted for the largest proportion. Most participants were rural household registration holders and married. Most participants had their own house and one or more children. One-third lived alone. Regarding the economy, more than one-third (37.6%) had a monthly income of less than RMB 1,000, and only 7.8% had a monthly income of more than RMB 4,000. Most of the participants (62.1%) had pension insurance. The proportion of people who had been diagnosed for 1–3 years was 45.1%. More than one-fifth had been diagnosed for more than 5 years. In terms of disease notification, There was a high proportion of participants (77.7%) informed their families and friends. More details are provided in Table 1.

**Table 1:** General characteristics of respondents (n = 319)

Variable	Groups	Frequency (n)	Percent (%)
**Sex**	Male	233	73.0
	Female	86	27.0
**Age (years)**	50–59	105	32.9
	60–69	116	36.4
	70–79	85	26.6
	≥ 80	13	4.1
**Education**	Primary school and below	212	66.5
	Junior high school	76	23.8
	Senior high school	24	7.5
	Junior college and above	7	2.2
**Household registration**	Urban	130	40.8
	Rural	189	59.2
**Marital status**	Married	202	63.3
	Divorced or widowed	104	32.6
	Unmarried	13	4.1
**Whether living alone**	Yes	107	33.5
	No	212	66.5
**whether having self-house**	Yes	270	84.6
	No	49	15.4
**Number of children**	0	18	5.6
	1	142	44.5
	2	116	36.4
	3	28	8.8
	4	6	1.9
	5	9	2.8
**Family economic stability**	Very unstable	24	7.5
	unstable	60	18.8
	general	90	28.2
	stable	89	27.9
	very stable	56	17.6
**Personal monthly income (RMB)**	<1000	120	37.6
	1001–2000	89	27.9
	2001–3000	44	13.8
	3001–4000	41	12.9
	>4000	25	7.8
**Endowment insurance**	Have	198	62.1
	Not have	121	37.9
**Monthly income and expenditure**	Insufficient	171	53.6
	Balance	87	27.3
	Surplus	61	19.1
**Length of diagnosis (years)**	<1	62	19.4
	1–3	144	45.1
	3–5	43	13.5
	>5	70	21.9
**Number of chronic diseases**	0	14	4.4
	1–3	143	44.8
	≥ 4	151	47.3
**Self-rated health**	Very poor	11	3.4
	Poor	47	14.7
	Fair	89	27.9
	Good	109	34.2
	Very good	63	19.7
**Whether informing friends and family of the condition**	Yes	248	77.7
	No	71	22.3

### Social support and HIV stigma of respondents

According to the total score of social support (Table 2), low level of social support accounted for 21.0% (67/319), a medium level of social support accounted for 78.1% (249/319), and a high level of social support accounted for only 0.9% (3/319). From the perspective of HIV stigma (Table 3), most participants had different degrees of HIV stigma. No, mild, moderate and severe perceived stigma accounted for 0.3% (1/319), 13.2% (42/319), 37.9% (121/319) and 48.6% (155/319), respectively. That is, only 0.3% (1/319) had no HIV stigma. A total of 13.2% (42/319) of older PWH had mild HIV stigma, while many older PWH (121/319, 37.9%) had moderate disease stigma. Nearly half of older PWH (155/319, 48.6%) had severe HIV stigma.

**Table 2:** Level of social support

Social support level	Score range	Frequency (n)	Percent (%)
Low	≤ 22	67	21.0
Medium	23–44	249	78.1
High	> 45	3	0.9

*Note*: The total score of social support ranges from 12 and 66

**Table 3:** Degree of HIV stigma

Degree of HIV stigma	Score range	Frequency (n)	Percent (%)
No	0	1	0.3
Mild	1–5	42	13.2
moderate	6–10	121	37.9
Severe	11–15	155	48.6

*Note*: The total score of HIV stigma ranges from 0 and 15

### Acceptable and preferred care models

Table 4 presents the acceptable and preferred care models of the participants. In terms of acceptable care models, the majority of older PWH chose home-based care (282/319, 88.4%). The number of people who could accept self-care was 88, accounting for 27.6% (88/319). Community-based care accounted for 26.0% (83/319), institutional care accounted for 20.7% (66/319), and mutual-aid care accounted for 11.9% (38/319). In terms of the preferred care model, the majority of older PWH preferred home-based care (232/319, 72.7%). Self-care ranked second among the preferred care models, accounting for 15.7% (50/319). Few people preferred community-based care (17/319, 5.3%), institutional care (16/319, 5.0%) and mutual-aid care (4/319, 1.3%), accounting for a low proportion.

**Table 4:** Acceptable and preferred care models of participants

	Groups	Frequency (n)	Percent (%)
Acceptable care models (multiple options)	home-based care	282	88.4
	self-care	88	27.6
	community-based care	83	26.0
	institutional care	66	20.7
	mutual-aid care	38	11.9
Preferred care model (single option)	home-based care	232	72.7
	self-care	50	15.7
	community-based care	17	5.3
	institutional care	16	5.0
	mutual-aid care	4	1.3

### Factors affecting care preference among older PWH

The results of the univariate analysis showed that literacy level, presence of a spouse, household registration, living alone or not, number of children, housing situation, monthly personal income, and level of HIV stigma had a statistically significant effect on the choice of preference for the mode of old-age care (*p* < .05). Then, the variables with statistical significance in univariate analysis were included in multinomial logistic regression. Table 5 shows the results of multivariate analysis. The total variance explained was 29%. The models were statistically significant (*p* < .05). Model 1 shows the comparison between self-care and home-based care. According to model 1, older PWH who lived alone preferred to choose self-care, and people who did not live alone were prone to choose home-based care. Compared with those who did not have their own house, older PWH with their own house were more likely to choose home-based care. In terms of monthly income, older PWH with higher income had higher willingness to choose self-care. Older PWH with a higher degree of HIV stigma tended to choose self-care. Model 2 presented the comparison between socialized care and home-based care. According to model 2, older PWH with a spouse were more willing to choose home-based care than those without a spouse. The number of children is a positive predictor of home-based care. Older PWH with more children had a preference for home-based care.

**Table 5:** Multinomial logistic regression analysis of care model preference

Variables	Model 1		Model 2	
	*OR (95% CI)*	*P*	*OR (95% CI)*	*P*
**Having a spouse**				
No	R		R	
Yes	1.80 (0.76–4.23)	0.180	0.29 (0.12–0.68)	0.004
**Household registration**				
Rural	R		R	
Urban	1.76 (0.82–3.79)	0.149	1.28 (0.55–2.97)	0.567
**Living alone**				
No	R		R	
Yes	3.66 (1.62–8.26)	0.002	0.82 (0.35–1.96)	0.669
**Having own house**				
No	R		R	
Yes	0.38 (0.16–0.91)	0.029	0.41 (0.16–1.03)	0.058
**Education**	1.28 (0.79–2.06)	0.317	1.26 (0.73–2.21)	0.402
**Number of children**	0.90 (0.58–1.38)	0.633	0.44 (0.27–0.72)	0.001
**Monthly income**	1.37 (1.02–1.85)	0.039	1.17 (0.83–1.64)	0.370
**Degree of HIV stigma**	2.59 (1.40–4.80)	0.002	0.82 (0.48–1.40)	0.459
**R** ^**2**^ **Nagelkerke**	0.29			

*Note*: R^2^ Nagelkerke is one type of pseudo R^2^ used as goodness-of-fit measure

*CI* = confidence interval; *OR* = odds ratio; R = reference category

## Discussion

Older PWH had the largest preference for home-based care. The preference for socialized care models (community-based care, institutional care and mutual-aid care) was relatively low. According to Cantor’s Hierarchical Compensatory Theory of Social Support [9], when older people need assistance, they usually turn to their spouses, children and other family members first. When family support is difficult to obtain, they turn to other informal support such as friends and neighbors. When this informal support is not available or the capacity of informal caregivers cannot meet their needs, they may turn to formal social care services such as institutional care and community-based care. Influenced by traditional Confucian culture, Chinese families mainly rely on intergenerational support to meet the care needs of older people [32, 33]. Hence the traditional home-based care occupies a dominant position in the pension security. One study [34] found that the choice of care model was affected by the fact and cost of pension, and older people were more willing to choose the care model with lower cost (such as home-based care). Home-based care is a traditional model in China, with high social identity and wide fact distribution. Older people can receive emotional support from their children and family members through home-based care. In response, social family structures should be strengthened in terms of legal protection, policy support and ideological foundations to urge children to fulfil their maintenance obligations.

In this study, self-care ranked second in the preference needs of care model, and a relatively large number of older PWH could accept self-care. Many older people had a desire to maintain independence [35]. Despite the availability of family support, older people seem to prefer to solve problems by themselves in order to avoid being a burden [36]. One study [37] found that older people’s willingness of care has obvious characteristics of independence and their identification with self-care has increased significantly, with the proportion rising from 0.11% to 1998 to 40.17% in 2017. After being diagnosed, many older PWH developed varying degrees of HIV stigma. They desire for independence and self-reliance [38]. From the perspective of the whole society, due to the reduction in family size, the acceleration of urbanization and increased labour mobility, the traditional Chinese home-based care function is weakening [39]. From the perspective of the disease, some older PWH suffered family discrimination from their spouses and children, and they were forced to live alone by being separated from their families [13, 14].

Older PWH had low acceptance and preference for community-based care, institutional care and mutual care, which is consistent with the willingness of ordinary older people [40, 41]. On the one hand, the cost of community-based care and institutional care is higher [42]. Most older PWH were from rural areas and had poor economic conditions, which made it difficult for them to afford the fees. According to Hierarchical Compensatory Theory of Social Support, older PWH may prefer home-based care when their children can provide care for them. It is only when home-based care is not available that older PWH will turn to socialized care services. Most older PWH had one or more children. Therefore, community-based and institutional care were not their first choice. The number of older PWH who chose mutual-aid care was few, which may be influenced by social participation and cognition [43]. That is, the lower the level of social participation, the lower the willingness to choose mutual-aid care. One study [29] indicated that older people who were willing to accept other people’s help had higher mutual-support needs. Mutual-aid care is a process of social interaction that requires communication and interaction. HIV stigma may affect the social integration of older PWH [44]. Older PWH were likely to suffer from social isolation as a result of less social participation and shrinking social networks [45]. In addition, as a new type of old-age care, older people generally lack knowledge about mutual-aid care [29]. Although the number of people choosing socialized old age care is relatively small, social development still requires diversified old age care to meet individualized old age needs. Hence the government can explore the AIDS medical and nursing care model or build nursing homes for infectious diseases to meet the institutional care needs of older PWH. Furthermore, based on the insufficiency of existing old age care resources and the demand for mutual-aid care of older PWH, the government can mobilize the social resources to explore a mutual-aid care model suitable for this group, and to solve the problem of old age care and security services for older PWH who are economically disadvantaged and living alone/empty nesting.

Our results showed that older PWH with a house, spouse and more children preferred home-based care. House is the material guarantee of living. One study indicated that older people who owned house property were more likely to choose home-based care [46]. Older people may prefer to stay in familiar homes and receive home-based care. Older married people were likely to rely on each other for support in their old care life which made them prefer home-based care. Studies [46, 47] pointed out that older people who had children preferred home-based care. Older people have lower income and depend more on their children. Raising children is a kind of old age care strategy in Chinese traditional culture. Support from children is the most important old-age security for most older people. Chinese Confucianism emphasizes that it is the responsibility and obligation of children to take care of their older parents [32, 33]. For older PWH, HIV infection is a major life change and blows to their later lives. They need the understanding, care and support from their partners, children and other family members. Therefore, it is necessary to consolidate and strengthen the function of the family in providing for older people, to carry out anti-discrimination interventions in the family, to reduce external discrimination, to urge children to fulfil their obligations of support, and to satisfy their home-based care needs.

We found that older PWH living alone, having higher personal monthly income and having higher HIV stigma were more likely to choose self-care. Older PWH, who lived alone, may suffer discrimination from family which led to low family support. Those older PWH actively or passively adapted to self-care and self-management in living alone, and their life independence is relatively strong. The higher the monthly income is, the better the economic independence and the more in line with the self-care model of “economic self-reliance”. ART helps older PWH maintain relatively healthy physical functions and they can take care of themselves well without other help. Due to the influence of HIV stigma, sexual shame, age discrimination and other factors [48, 49], older PWH are usually socially isolated and report heavy intrinsic HIV stigma [44]. People who were isolated had lower levels of assistance and lower perceptions of support availability and adequacy [14]. Self-care may be a strategy adopted by older PWH to cope with social discrimination. Based on the tendency of older PWH to self-care, the government could provide employment services for older PWH who are willing to work, create more re-employment jobs, enhance their economic self-reliance, and promote their participation in social activities, so as to enable them to resume their normal social life.

### Strengths and limitations

This study used a cross-sectional design, so causal inferences cannot be obtained. Due to limited time and resources, the data were collected at one city in China, so the representativeness of the study was limited. In the future, multi-center large-scale research can be conducted in conjunction with other institutions. This study lacked a theoretical or conceptual framework. Questionnaires were collected by self-reporting, so it is possible to report recall bias, especially social desirability bias, in that participants may report results based on social expectations rather than their actual thoughts. Despite these limitations, our work is novel, as it may be the first study to examine the preference for different care models among older PWH.

## Conclusions

Older PWH were more inclined to choose home-based care. Self-care ranked second among the preferred care models. The number of those willing to receive institutional care, community-based care and mutual-aid care accounted for a relatively large proportion, but few people preferred these models. Having a house, a spouse and number of children were associated with preference for home-based care. Living alone, monthly income and HIV stigma were associated with preference for self-care. China should incorporate older PWH care into the national social security system and measures should be taken to allocate the old-age resources reasonably to better meet the care needs of older PWH. More attention should be given to reducing internalized HIV stigma, strengthening social support, exploring diversified aging care models and improving quality of life.

### Electronic supplementary material

Below is the link to the electronic supplementary material.

**Additional file 1: Supplementary Material 1**. shows the questionnaire on preference for care models among older people living with HIV

## Data Availability

The datasets used and analysed during the current study are available from the corresponding author on reasonable request.

## Abbreviations

PWH: People living with HIV
older PWH: Older people living with HIV
ART: Antiretroviral Therapy

## References

[1] Wei Hui LB, Guang Guang Hua (2021). Research progress of AIDS epidemic characteristics in elderly population in China. Appl Prev Med.

[2] Negredo E, Back D, Blanco J-R, Blanco J, Erlandson KM, Garolera M, Guaraldi G, Mallon P, Moltó J, Serra JA (2017). Aging in HIV-Infected subjects: a new scenario and a New View. Biomed Res Int.

[3] Sánchez-Conde M, Díaz-Alvarez J, Dronda F, Brañas F (2019). Why are people with HIV considered older adults in their fifties?. Eur Geriatr Med.

[4] Blanco JR, Jarrín I, Vallejo M, Berenguer J, Solera C, Rubio R, Pulido F, Asensi V, del Amo J, Moreno S (2012). Definition of advanced age in HIV Infection: looking for an age cut-off. AIDS Res Hum Retroviruses.

[5] Tavoschi L, Gomes Dias J, Pharris A (2017). New HIV diagnoses among adults aged 50 years or older in 31 European countries, 2004-15: an analysis of surveillance data. Lancet HIV.

[6] Smit M, Brinkman K, Geerlings S, Smit C, Thyagarajan K, de Sighem Av F, Hallett TB (2015). Future challenges for clinical care of an ageing population infected with HIV: a modelling study. Lancet Infect Dis.

[7] Na H (2021). Research Progress in the epidemiology of HIV/AIDS in China. China CDC Weekly.

[8] Porter L (2017). Older people with HIV enter uncharted territory. Nurs Older People.

[9] Cox LE, Brennan-Ing M (2017). Medical, Social and Supportive Services for older adults with HIV. Interdiscip Top Gerontol Geriatr.

[10] Sun-Suslow N, Pasipanodya E, Morgan E, Kohli M, Serrano V, Letendre S, Jeste DV, Moore DJ (2020). Social support moderates D-dimer and self-rated successful aging within people with HIV and older adults. J Behav Med.

[11] Gannon BN, Stacciarini J-MR (2016). Review of the literature: a rural-urban comparison of Social Networks of older adults living with HIV. J Assoc Nurses AIDS Care.

[12] Grodensky CA, Golin CE, Jones C, Mamo M, Dennis AC, Abernethy MG, Patterson KB (2015). I should know better: the roles of relationships, spirituality, disclosure, stigma, and shame for older women living with HIV seeking support in the South. J Assoc Nurses AIDS Care.

[13] Quinn KG, Murphy MK, Nigogosyan Z, Petroll AE (2020). Stigma, isolation and depression among older adults living with HIV in rural areas. Ageing Soc.

[14] Brennan-Ing M, Seidel L, Karpiak SE (2017). Social Support Systems and Social Network Characteristics of Older Adults with HIV. Interdiscip Top Gerontol Geriatr.

[15] Naudet D, De Decker L, Chiche L, Doncarli C, Ho-Amiot V, Bessaud M, Alitta Q, Retornaz F (2017). Nursing home admission of aging HIV patients: challenges and obstacles for medical and nursing staffs. Eur Geriatr Med.

[16] Shippy RA, Karpiak SE (2005). The aging HIV/AIDS population: fragile social networks. Aging Ment Health.

[17] Nobre NR, Kylmä J, Kirsi T, Pereira M (2016). Social networks of older adults living with HIV in Finland. AIDS Care.

[18] Foebel AD, Hirdes JP, Boodram C, Lemick R, Tai JW, Comeau RL (2016). Comparing the care needs of people living with and without HIV in Canadian home and long-term care settings. Can Commun Dis Rep.

[19] Akintola O, Hangulu L (2014). Infection control in home-based care for people living with HIV/AIDS/TB in South Africa: an exploratory study. Glob Public Health.

[20] Crock EA, Miller C, McKenzie R, Burk N, Frecker J, Hall JE, Ramirez OM (2017). Emerging needs of people living with HIV receiving community-based nursing in an Australian setting. J Assoc Nurses AIDS Care.

[21] Koehn K, Burgess H, Lyndon S, Lu M, Ye M, Hogg RS, Parashar S, Barrios R, Salters KA (2021). Characteristics of older adults living with HIV accessing home and community care services in British Columbia, Canada. AIDS Care.

[22] Seidel L, Karpiak SE, Brennan-Ing M (2017). Training senior service providers about HIV and aging: evaluation of a multiyear, multicity initiative. Gerontol Geriatr Educ.

[23] Buchanan RJ, Wang S, Huang C (2002). Profiles of nursing home residents with HIV. J Health Care Poor Underserved.

[24] MacDowell NM (1989). Willingness to provide care to AIDS patients in Ohio nursing homes. J Community Health.

[25] Fogarty TE, Gentry D, Lehrman SE (1997). Defining the challenges of providing long-term care: the case of the nursing home industry’s response to the AIDS epidemic. J Aging Soc Policy.

[26] Miller SC, Cai S, Daiello LA, Shireman TI, Wilson IB (2019). Nursing home residents by human immunodeficiency Virus Status: characteristics, Dementia diagnoses, and antipsychotic use. J Am Geriatr Soc.

[27] S X: Theoretical basis and research application of Social Support rating scale. J Clin Psychiatry 1994(2):98–100.

[28] Li L, Guo Y (2010). Validity and reliability of simplified HIV stigma Scale in multi-ethnic areas. Chin Mental Health J.

[29] VanVoorhis CW. Morgan BLJTiqmfp: Understanding power and rules of thumb for determining sample sizes. 2007, 3(2):43–50.

[30] Zou Xiaojuan ZY (2010). Application and significance of preliminary investigation in clinical epidemiological investigation. J Hubei Univ Traditional Chin Med.

[31] Jiang Xiangqun ZY (2006). Social elderly care: problems and challenges. Beijing Observation.

[32] Zhang L, Han Y, Ma Y, Xu Z, Fang Y (2021). Eastern perspectives on roles, responsibilities and filial piety: a case study. Nurs Ethics.

[33] Liu J, Bern-Klug M (2016). I should be doing more for my parent: Chinese adult children’s worry about performance in providing care for their oldest-old parents. Int Psychogeriatr.

[34] Jia Y (2020). Effect of factual pension and pension cost on the willingness of the Elderly to choose the pension model: evidence from 915 Elderly people in Shaanxi Rural areas. Areal Res Dev.

[35] Abdi S, Spann A, Borilovic J, de Witte L, Hawley M (2020). Correction to: understanding the care and support needs of older people: a scoping review and categorisation using the WHO international classification of functioning, disability and health framework (ICF). BMC Geriatr.

[36] Du Q, Gong N, Hu Q, Chen G, Xie J, Luo L, Cheng Y, Zhang M (2022). Why do older adults living alone in cities cease seeking assistance? A qualitative study in China. BMC Geriatr.

[37] Liang H (2020). The change of the Elderly’s willingness to choose the patterns of old-age support in Large City(1998–2017): an empirical analysis of Guangzhou. Popul South China.

[38] Schrimshaw EW, Siegel K (2003). Perceived barriers to social support from family and friends among older adults with HIV/AIDS. J Health Psychol.

[39] Feng Z, Glinskaya E, Chen H, Gong S, Qiu Y, Xu J, Yip W (2020). Long-term care system for older adults in China: policy landscape, challenges, and future prospects. Lancet.

[40] Wang H, Guan Y, Hu R, Bragg F, Yu M, Zhong J (2022). Willingness for community-based and institutional eldercare among older adults: a cross-sectional study in Zhejiang, China. BMJ Open.

[41] Yao K-R, Yin X-H, Luo Q, Tang X, Tan X-Z (2022). Factors influencing the mutual-support willingness and needs among the rural elderly in Hunan Province, China: a cross-sectional study. BMC Health Serv Res.

[42] Liu Z-W, Yu Y, Fang L, Hu M, Zhou L, Xiao S-Y (2019). Willingness to receive institutional and community-based eldercare among the rural elderly in China. PLoS ONE.

[43] Tao W, Zhang S, Zhang L, Xue L, Liu Y, Zheng Y, Gao S. The influencing factors of willingness to participate in community mutual aid pension among the elderly in community. China Nurs Manage, 20(4):535–9.

[44] Brennan-Ing M (2019). Diversity, stigma, and social integration among older adults with HIV. Eur Geriatr Med.

[45] Rueda S, Law S, Rourke SB (2014). Psychosocial, mental health, and behavioral issues of aging with HIV. Curr Opin HIV AIDS.

[46] Xing Y, Pei R, Qu J, Wang J, Zhou H, Wang Z, Yan W, Sun X, Sun T, Li L (2018). Urban-rural differences in factors associated with willingness to receive eldercare among the elderly: a cross-sectional survey in China. BMJ Open.

[47] Gustafson K, Baofeng H (2014). Elderly care and the one-child policy: concerns, expectations and preparations for elderly life in a rural Chinese township. J Cross Cult Gerontol.

[48] Kiplagat J, Mwangi A, Chasela C, Huschke S (2019). Challenges with seeking HIV care services: perspectives of older adults infected with HIV in western Kenya. BMC Public Health.

[49] Xu Y, Lin X, Chen S, Liu Y, Liu H (2018). Ageism, resilience, coping, family support, and quality of life among older people living with HIV/AIDS in Nanning, China. Glob Public Health.

